# Porous Adsorption Materials for Carbon Dioxide Capture in Industrial Flue Gas

**DOI:** 10.3389/fchem.2022.939701

**Published:** 2022-06-29

**Authors:** Hongxue Zeng, Xinghong Qu, Dong Xu, Yang Luo

**Affiliations:** ^1^ Zhejiang Tongji Vocational College of Science and Technology, Hang Zhou, China; ^2^ College of Geomatics and Municipal Engineering, Zhejiang University of Water Resources and Electric Power, Hang zhou, China; ^3^ Empa, Swiss Federal Laboratories for Materials Science and Technology, ETH Domain, Dübendorf, Switzerland

**Keywords:** carbon capture and storage, porous adsorption materials, industrial waste gas, adsorption mechanism, carbon dioxide

## Abstract

Due to the intensification of the greenhouse effect and the emphasis on the utilization of CO_2_ resources, the enrichment and separation of CO_2_ have become a current research focus in the environment and energy. Compared with other technologies, pressure swing adsorption has the advantages of low cost and high efficiency and has been widely used. The design and preparation of high-efficiency adsorbents is the core of the pressure swing adsorption technology. Therefore, high-performance porous CO_2_ adsorption materials have attracted increasing attention. Porous adsorption materials with high specific surface area, high CO_2_ adsorption capacity, low regeneration energy, good cycle performance, and moisture resistance have been focused on. This article summarizes the optimization of CO_2_ adsorption by porous adsorption materials and then applies them to the field of CO_2_ adsorption. The internal laws between the pore structure, surface chemistry, and CO_2_ adsorption performance of porous adsorbent materials are discussed. Further development requirements and research focus on porous adsorbent materials for CO_2_ treatment in industrial waste gas are prospected. The structural design of porous carbon adsorption materials is still the current research focus. With the requirements of applications and environmental conditions, the integrity, mechanical strength and water resistance of high-performance materials need to be met.

## 1 Introduction

**FIGURE 1:**
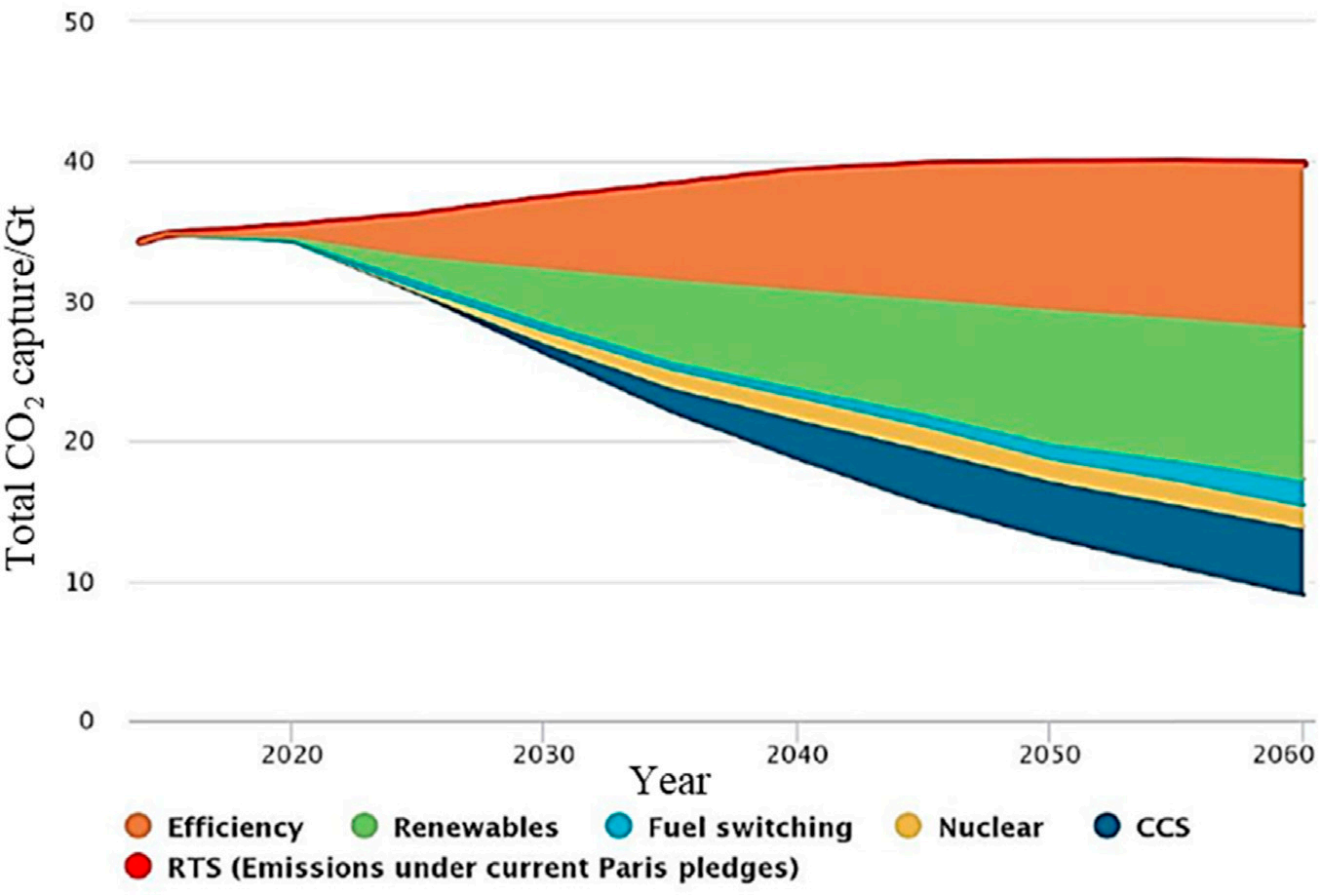
The contribution rate of different technologies to carbon emission reduction in the 2DS scenario (IEA, 2017).

Due to the massive consumption of fossil fuels (coal, oil, natural gas), greenhouse gases cause global temperature rise, and it is imminent to curb global warming. As the 195 member states of the United Nations Framework Convention on Climate Change reached the Paris Agreement at the Paris Climate Change Conference, the agreement established a global action plan to limit the rise in global average temperature this century to 2°C of pre-industrial revolution levels or lower. And try to control the global temperature rise below 1.5°C to avoid the crisis brought by climate change, which also builds a bridge between today’s policy and the climate goals by the end of this century (Lesnikowski et al., 2017). With the goal of controlling the temperature rise of 2°C, both the Intergovernmental Panel on Climate Change (IPCC) and the International Energy Agency (IEA) have emphasized the important role of carbon capture and storage (CCS) technology in achieving long-term high-efficiency carbon dioxide emission reduction. Carbon capture and storage (CCS) is considered, one of the effective measures to cope with the challenge of climate change. It has irreplaceable advantages in reducing carbon dioxide emissions. CCS has strong adaptability to the existing energy system, stable operation, and can achieve substantial emission reduction of carbon dioxide in the power system. It is essential to ensure energy security and achieve sustainable development (Bui et al., 2018). CCS is the only way for coal-intensive industries such as coal chemical industry, cement, steel and oil refineries to achieve substantial carbon dioxide emissions reductions. CCS and renewable energy can form complementary technologies to achieve decarbonization goals (Haszeldine, 2009). Carbon dioxide direct air capture technology (DAC) and biomass energy CCS technology (BECCS) can achieve large-scale harmful carbon emissions (Change, 2014; Ma et al., 2021). The combination of CCS and steam methane reforming can obtain “blue hydrogen”, also called carbon-neutral hydrogen, which promotes the transformation of the energy system to carbon-neutral (Davison, 2009). However, the current promotion of CCS still has a high degree of uncertainty, and its progress depends on the degree of reduction in energy consumption and cost of the whole chain of CCS, the cost competition between CCS and other low-carbon technologies. The gain of carbon capture benefits throughout the life cycle of CCS and other factors. Figure 1 shows the “2 °C scenario (2DS)” energy technology route given by IEA in the “Energy Technology Outlook 2017” (Orr, 2009). The model predicts that from 2015 to 2060, the cumulative carbon emission reduction achieved through CCS technology will be 14%. In 2050 and 2060, the carbon dioxide emission reduction through CCS will need to reach 4.2 Gt and 4.9 Gt, respectively. In the below 2°C scenario (B2DS) route predicted by the IEA, by 2060, the cumulative carbon emission reduction contribution contributed by CCS needs to reach 32%. How to efficiently capture the carbon dioxide in the flue gas is a favorable way to effectively control the increase of greenhouse gases.

CCS is a unified whole of the organic connection of carbon dioxide capture, transportation, storage, and utilization. The technology involved in the entire process chain is diverse and complex, and the development stages of different technologies are not the same. According to the technical characteristics of the carbon dioxide separation process, the carbon dioxide capture technology of thermal power plants is usually divided into most mature carbon capture technology is the post-combustion carbon dioxide capture technology of chemical absorption based on alcohol amine absorbents (Gibbins and Chalmers, 2008). The post-combustion carbon dioxide capture technology of chemical absorption based on alcohol amine absorbents is currently the most mature carbon capture technology. Large-scale commercial operations were achieved in the United States and Canada in 2014 and 2017 (Lipponen et al., 2017). In addition, MTR (Membrane Technology and Research) successfully developed for the first time a polymer membrane (Polaris Membrane) that can be used in commercial applications to separate carbon dioxide from syngas (Kniep et al., 2017). Post-combustion capture is a technology that captures carbon dioxide from the flue gas after the boiler is burned. It separates carbon dioxide with a lower partial pressure from high-concentration nitrogen and a small amount of oxygen and water vapor mainly used in coal-fired, oil-fired and gas-fired power plants or carbon dioxide capture in natural gas combined cycles. This technology has little impact on the existing power plant production process. The energy consumption of carbon dioxide capture can be controlled by adjusting the carbon dioxide separation efficiency or flue gas flow. In addition, this technology can be used in many industrial processes (such as the cement industry and the steel industry). However, due to the low concentration of carbon dioxide and the low partial pressure of carbon dioxide in the flue gas, the CO_2_ capture material after combustion needs to have good CO_2_ adsorption and separation performance under low pressure (Kanniche et al., 2010). Based on the characteristics of low CO_2_ partial pressure of power plant flue gas, post-combustion capture is currently the most common capture method. The capture mode can be divided into the absorption method, adsorption method, membrane separation method, cryogenic distillation method, etc., Compared with the post-combustion capture technology, the integrated gasification combined cycle (IGCC) pre-combustion capture and oxygen-enriched combustion technology has a lower technical readiness index, and large-scale commercial applications have not yet been realized.

**FIGURE 2:**
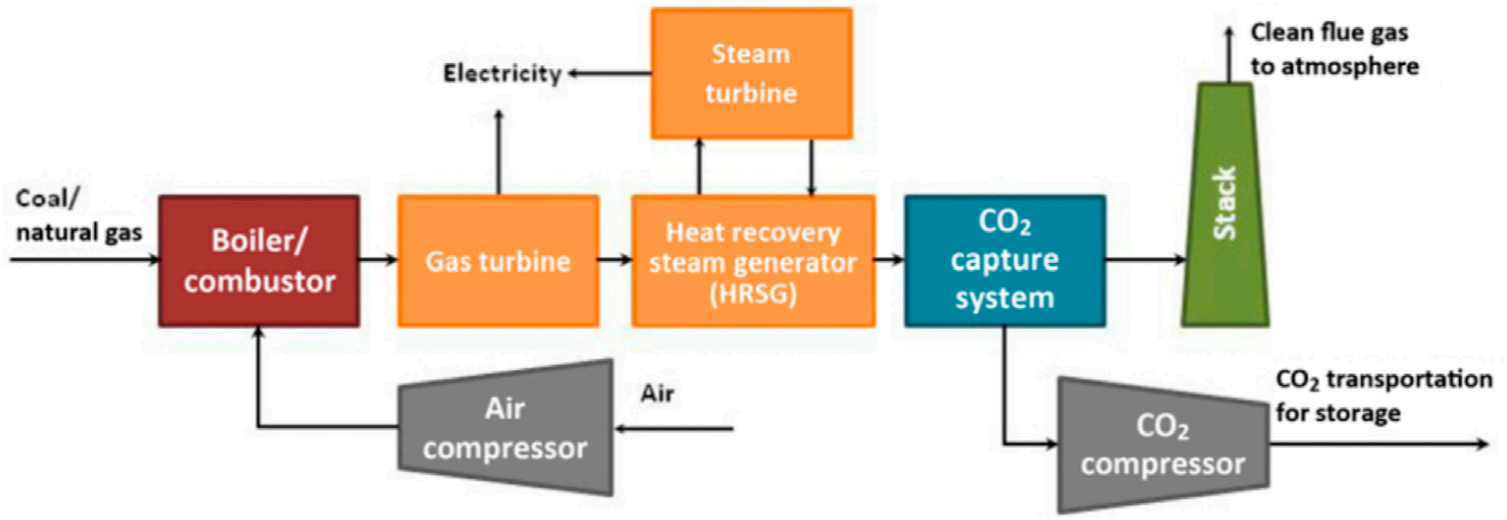
Schematic diagram of the combustion CO_2_ capture device of a coal-fired power plant (Zaman and Lee, 2013).

The post-combustion capture system is shown in Figure 2. The post-combustion capture process can be divided into the following three steps: 1) Pre-purification of flue gas, boiler flue gas is purified by denitration, dust removal, and desulfurization to meet the requirements of CO_2_ separation equipment; 2) CO_2_ capture, flue gas enters CO_2_ absorption/adsorption device (such as MEA absorption tower) realizes the removal of CO_2_, and the flue gas (mainly N_2_, water vapor) that does not contain (or contains low concentration) CO_2_ is discharged through the chimney; 3) Absorbent/adsorbent Regeneration, the CO_2_-rich absorbent or adsorbent releases high-purity CO_2_ to achieve regeneration. It is worth noting that the CO_2_ content in the flue gas during post-combustion capture is the lowest among the three capture technologies, only 3–20 vol% (Ebner and Ritter, 2009). Therefore, this technology has very high requirements for absorbents/adsorbents. Since the temperature range of the synthesis gas for capturing CO_2_ before combustion in IGCC is 250–400°C (Xiao et al., 2011), a medium temperature adsorbent should be used. The post-combustion capture system in coal-fired power plants mainly adopts the solvent absorption method based on alcohol amine solution. However, the traditional solvent absorption method has shortcomings such as high energy consumption and high corrosiveness, and it is urgent to develop a more cost-effective absorbent/adsorbent (Subha et al., 2015; Vega et al., 2020; Kearns et al., 2021). Common CO_2_ capture materials include activated carbon, zeolite, molecular sieves, metal oxides, and metal-organic framework materials (Leperi et al., 2019; Pardakhti et al., 2019; Omodolor et al., 2020; Lin et al., 2021). The main criteria for a better adsorbent material should include the following aspects: higher specific surface area, better adsorption selectivity, good adsorption performance and cycle stability. This review discusses how to optimize porous adsorption materials based on the treatment of CO_2_ in industrial waste gas around the above three dimensions. And summarized a series of problems currently faced, and looked forward to the future research direction.

## 2 Adsorption Mechanism of Carbon Dioxide

The adsorption method uses an adsorbent to selectively adsorb a specific gas to achieve the purpose of separation. The complete adsorption process includes two steps: adsorption and desorption. Periodic adsorption and desorption are used to complete the concentration of carbon dioxide and the regeneration of adsorbent materials. The continuous operation can be achieved by parallel adsorption towers (Shen et al., 2017; Li et al., 2020a). According to the different adsorption mechanism, the adsorption method can be divided into physical and chemical adsorption. Physical adsorption refers to the combination of adsorbate molecules and the surface of the adsorbent through microscopic forces (Coulomb force and Van der Waals force) to form stable adsorption without forming chemical bonds. When the adsorbed gas contacts the adsorbent, due to the attraction of the adsorbent surface, the adsorbed gas will form an adsorption layer on the surface of the adsorbent. This adsorption layer is called the adsorption phase. The density of the adsorption phase is much greater than thegas density. The remaining gravitational force on the surface and the gravitational force of the gas molecules are combined, and the adsorption affinity produced by the mutual combination is called the adsorption force. There are generally three sources of adsorption force: 1) Van der Waals force including repulsive force and attractive force prevailing between atoms and molecules; 2) Electrostatic Coulomb force between charged particles; 3) Due to the induction of the permanent dipole moment, the adjacent molecules produce charge displacement, which causes the induced polarization and produces the induced force of the induced dipole moment. The desorption of adsorbed gas molecules on the surface of the framework material can be completed under low energy consumption, and the reuse of the framework material is realized, which is the most significant advantage of physical adsorption. Physical adsorption adsorbent has good regeneration performance, but its selectivity is poor, and the adsorption capacity is low at high temperatures. Chemical adsorption refers to the formation of chemical bonds between gas molecules and the surface of the adsorbent to bond them together.

The adsorbent has a high adsorption capacity and good selectivity, but its desorption temperature is high, and regeneration consumes a lot of energy. Regarding the renewal and recycling of adsorbent materials in the process of adsorbing carbon dioxide by the adsorption method, the adsorption process generally includes the following: temperature swing adsorption (TSA), regenerative pressure swing adsorption (PSA), and electric swing adsorption (ESA). TSA relies on temperature changes to achieve carbon dioxide adsorption separation and regeneration of adsorbent materials and relies on pressure changes to achieve carbon dioxide separation and adsorption materials, while PSA and ESA change the coupling effect of temperature and pressure, as shown in Table 1.

**TABLE 1:** Common adsorption carbon capture processes.

Adsorption process	Principle	Advantage	Disadvantage
PSA	Change system pressure	Simple system and high stability	Pressurization is required before adsorption; air intake energy consumption is large; regeneration is not complete
TSA	Different temperature, different adsorption capacity	High adsorption efficiency and thorough regeneration	Large thermal inertia; long regeneration time; hot gas medium is easy to dilute the purity of gas produced; large energy consumption
Electric swing adsorption (ESA)	The essence is temperature swing adsorption, desorption electric heating	Energy efficiency; high heating rate	Immature process
Vacuum swing adsorption (VSA)	Change system pressure (Vacuum)	Suitable for low pressure, large scale applications	High capture energy consumption

Pressure swing adsorption technology is a fixed-bed separation technology. The separation or purification of carbon dioxide and the regeneration of adsorption materials are carried out by changing the adsorption pressure under constant temperature conditions. Generally speaking, at the same temperature, the equilibrium adsorption capacity increases as the pressure increases; when the pressure decreases, the equilibrium adsorption capacity also decreases. Therefore, adsorption can be carried out at high pressure, and desorption can be carried out at low pressure. Pressure swing adsorption uses periodic pressure changes to make the adsorption material adsorb and separate carbon dioxide, this is pressure swing adsorption (Liu et al., 2011; Shen et al., 2017; Wawrzyńczak et al., 2019). The general pressure swing adsorption operation process indicates high-pressure adsorption and atmospheric desorption. Nowadays, vacuum pressure swing has been developed, which means adsorption under normal pressure or slightly higher than normal pressure and desorption under vacuum conditions. Pressure swing adsorption achieves adsorption and desorption by periodically changing the pressure,, including adsorption at high pressure, and then achieves desorption by reducing the pressure or vacuuming. Electric swing adsorption is to directly heat the adsorbent particles to increase the temperature, so as to achieve the purpose of desorption. This method has the advantages of fast desorption speed and high energy utilization, but this method usually requires the adsorbent material to have good conductivity (Grande et al., 2009; Grande et al., 2010; Zhao et al., 2018). Under certain conditions, the adsorbent selectively adsorbs CO_2_ in the flue gas. It then changes certain conditions (such as temperature, pressure, etc. ) to desorb CO_2_ from the adsorbent to achieve the purpose of CO_2_ separation. Rigid solid adsorbent materials capture gas molecules through physical adsorption and are one of the most potential carbon dioxide capture and separation candidates..

The temperature swing adsorption is simple to operate and easy to implement, but it has higher requirements for the thermal conductivity of the adsorbent material. Temperature swing adsorption relies on the characteristic that the adsorption capacity of carbon dioxide changes with temperature. Under normal circumstances, when the temperature is at the same pressure, the lower the temperature, the equilibrium adsorption capacity increases as the temperature decreases; when the temperature increases, the equilibrium adsorption capacity decreases. Therefore, it can be adsorbed at low temperature and desorbed at high temperatures. The adsorption material can be circulated to adsorb and separate carbon dioxide through periodic temperature changes, demonstrating temperature swing adsorption (Zhao et al., 2019). When capturing carbon dioxide in flue gas, for some adsorption materials with a strong affinity for carbon dioxide, it is challenging to complete the regeneration of the adsorption material if only by changing the pressure. Therefore, temperature swing adsorption is required. There are two types of temperature swing adsorption. One is to use a heating medium to heat, such as purging the adsorption material with high-temperature inert gas, or using the adsorption material’s resistance for electrical heating, and the other is indirect heating, such as the use of coils, jackets, etc. Generally speaking, temperature swing adsorption products have a high recovery rate and low loss. Still, the cycle is long, the investment is enormous, the energy consumption is high, and the service life of the adsorbent is not long. The pressure swing adsorption cycle is short and the adsorbent utilization rate is high, but the recovery rate is low. Therefore, the independent use of temperature or pressure swing methods to recycle molecular sieves has certain defects (Choi et al., 2016; Jiang et al., 2018).

Table 2 lists the main carbon emission sources to which adsorption methods can be applied. Traditional post-combustion carbon capture is the process of separating CO_2_ from the flue gas formed after the combustion of fuel and air. Among them, the type of fuel and excess air coefficient determine the total gas volume and dry basis CO_2_ concentration of flue gas, ranging from 3% to 4% of natural gas combined cycle (NGCC) to pulverized coal boiler power station and integrated coal gasification combined cycle (IGCC) ranging from 14%. Pre-combustion carbon capture requires the conversion of fuel (coal, heavy oil, residual carbon, etc.) into syngas or reformed gas through steam reforming or partial oxidation, decarbonization, and combustion in a gas turbine to generate electricity, or CO through water-gas shift (WGS) reaction into CO_2_ and H_2_, followed by decarbonization. For the latter case, the CO_2_ concentration in the shift gas can be as high as 60%, so it is easier to separate CO_2_ and simultaneously obtain high-purity H_2_ as an energy carrier or chemical raw material. It should be noted, however, that the initial fuel gasification/reforming process is expensive to operate. In addition to the above two typical capture technologies, adsorption methods can also be applied to carbon capture from emission sources such as steel mills, cement plants, biogas, and flare gas. Recently, adsorption-based direct air capture (DAC) has also received more and more attention as a potential negative emission technology.

**TABLE 2:** Main emission sources of common adsorption carbon capture (Jahandar Lashaki et al., 2019).

Component	Coal burning	Smoke					Biogas		Syngas		Air
		Waste incineration	IGCC	NGCC	Cement kiln	Household waste	Agricultural waste	Agri-Food/Industrial Waste	Wabash River Coal Gasification Project	Tampa IGCC Power Station	
O_2_ (vol/vol)	6	7–14	12	14	7	0–1	<0.5				20.946
N_2_ (vol/vol)	76	Balance gas	66	76	59	0–5	0–1			3.3	78.084
CO_2_ (vol/vol)	11	6–12	7	3	19	34–38	19–33	26	14.9–17.1	14.4	0.041
H_2_O(vol/vol)	6	10–18	14	6	13	6	6	6		0.3	
Ar (vol/vol)	1	1	1	1	1					0.9	0.934
CH_4_(vol/vol)						50–60	60–75	68	1–2.2	0.1	
H_2_ (vol/vol)									32.3–34.4	38.3	
CO(vol/vol)									42.2–46.7	42.7	
SO_2_(ppm, parts per million)	300–5000	200–1500	10–200		5–1200						
NO_x_ (ppm)	500–800	200–500	10–100	10–300	100–1500						
H_2_S(ppm)						100–900	3000–10,000	400	17–107	200	
COS(ppm)									9–162	10	

The adsorption method to capture carbon dioxide has the outstanding advantages of simple process, mild operating conditions, extensive operating flexibility, sizeable operating temperature range, low operating cost, stable performance, and no corrosion and pollution, suggesting a carbon dioxide capture method with excellent development potential. The post-combustion capture process of flue gas is characterized by its high temperature, atmospheric pressure, and low CO_2_ concentration. Therefore, the physical adsorption method greatly affected by the CO_2_ concentration cannot effectively separate CO_2_. In contrast, the solvent absorption method requires the gas to be cooled to a specific temperature first, which has a significant energy loss and high energy consumption for regeneration of the absorption solvent. It is corrosive to equipment (Rochelle, 2009). If a solid adsorbent is used to capture CO_2_ in this temperature range, the above problems can be effectively avoided.

## 3 Research Direction of CO_2_ on Porous Adsorption Materials

### 3.1 Specific Surface Area of the Porous Adsorbent

To continuously meet the demand for a high specific surface area of adsorbent materials, under the inspiration of nature, researchers have designed and synthesized a wealth of porous materials, which has promoted the rapid development of porous materials. In order to facilitate the study and discussion of it, according to the composition of the porous material framework, the carbon dioxide porous adsorbent materials are roughly divided into two categories: 1) inorganic porous materials, such as zeolites, mesoporous silica, and metal oxide and 2) inorganic-organic hybrid porous materials, such as metal organic frameworks (MOFs) (Xu et al., 2016; Senker, 2018; Zhao et al., 2020). These two types of materials have different structural characteristics and therefore have distinct material properties. The comparison of their properties is shown in Table 3. It can be seen that although the pore size and crystal type of the two porous adsorption materials are more common, the chemical stability and thermal stability are still quite different, which limits their application environment (including temperature and pH, etc.). MOFs materials are more diverse in chemistry and structure than zeolite.

**TABLE 3:** Comparison of the performance of two common porous materials.

Characteristic	Zeolites	MOFs	Carbon material	Metal oxide
Pore size	Microporous, mesoporous, macroporous	Microporous, mesoporous, macroporous	Microporous, mesoporous, macroporous	Microporous, mesoporous, macroporous
Crystallinity	Typical	Typical	/	/
Thermal stability	High	Low-medium	Low-medium	Low
Chemical stability	Good (except acid and alkali environment)	Poor-good	Good	Poor
Chemical diversity	Medium	Very high	Low-medium	Poor
Structural diversity	High	Very high	Very high	Poor

**FIGURE 3:**
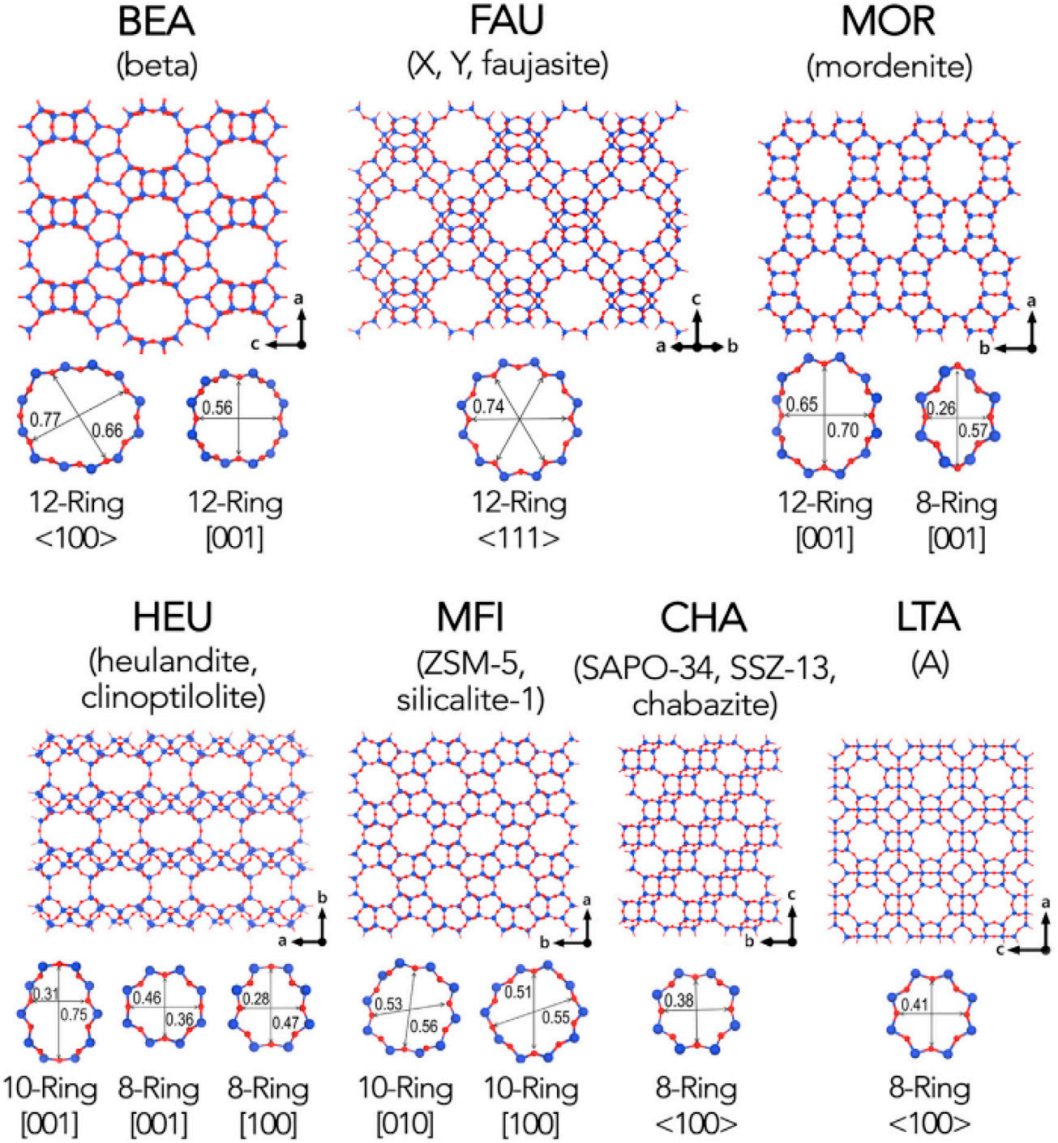
Common framework types of zeolite molecular sieve (Li et al., 2017). The blue sphere represents the T atom, and the red sphere represents the oxygen atom.

Zeolite is an aluminosilicate with a porous crystal structure and a TO_4_ (T is Si or Al) tetrahedral periodic structure. Its unique molecular sieve effect is widely used in gas separation processes. In the framework of a molecular sieve, primary structural units such as silicon-oxygen tetrahedron (SiO_4_) and aluminum-oxygen tetrahedron (AlO_4_) combine to form a variety of multi-ring secondary structural units through shared oxygen atoms (Figure 3). Oxygen bridge bonds connect the multiple rings to form molecular sieve materials with different types of three-dimensional network structures (Li et al., 2017). The most classic molecular sieve structures are LTA and FAU. As a typical physical adsorbent material, zeolite has a robust electrostatic force between carbon dioxide molecules and alkali metal ions in the process of CO_2_ capture (Krishna and Van Baten, 2007), the adsorption rate of carbon dioxide is very speedy, and it often reaches saturation within a few minutes. Zeolite has a unique pore structure, and the inside of the crystal has a specific electrostatic force and dispersion force, which gives zeolite selective adsorption characteristics (Krishna and van Baten, 2020; Zhou et al., 2021). However, its adsorption performance is affected by adsorption temperature and pressure. As the adsorption temperature increases, the CO_2_ adsorption capacity of the zeolite decreases, and as the CO_2_ partial pressure increases, the CO_2_ adsorption capacity of the zeolite increases. As shown in Table 4, molecular sieves can be divided into A-type molecular sieves, X-type molecular sieves, Y-type molecular sieves, ZSM series molecular sieves (Siriwardane et al., 2001), and so on, according to the crystal framework structure of molecular sieves. Among these molecular sieves, A-type, X-type and Y-type molecular sieves are the most used for carbon dioxide adsorption.

**TABLE 4:** Specifications of commonly used molecular sieves.

Parameter	3A	4A	5A	10X	13X
Extra-framework cations	2/3K_2_O	Na_2_O	3/4CaO	4/5CaO	Na_2_O
	1/3Na_2_O		1/4 Na_2_O	1/5 Na_2_O	
Si/Al ratio	2	2	2	2.6–3.0	2.6–3.0
Effective pore size (nm)	0.3	0.4	0.5	0.9	1.0
Main adsorption	Ethylene	Short carbon alkanes	Normal/Isomeric Alkanes	Aromatic hydrocarbons	Carbon dioxide

In addition, the strong cation exchange properties make zeolite easy to modify, and the cost of zeolite is low, which is very suitable for large-scale production applications (Thang et al., 2014). Pham et al. (Pham et al., 2016) studied the CO_2_ capture performance of synthetic nano-zeolites by temperature swing adsorption (TSA). The results show that under the conditions of 20°C and 1bar, the adsorption capacity of nano-zeolite for CO_2_ is 4.81 mmol/g. Using ideal adsorption solution theory (IAST), the selective adsorption of nano-zeolite is calculated to be 18.65. The CO_2_ removal rate was maintained above 88% through ten cycles of adsorption-desorption regeneration experiments. Zeolite has strong hydrophilicity, and the apparent decrease of CO_2_ adsorption performance under humid conditions limits its further development. In zeolite materials, factors such as the composition and structure of the framework, the replacement of cations, the purity of the zeolite, the size and distribution of the pore size and other factors will affect the adsorption performance of the zeolite molecular sieve to adsorb carbon dioxide.

**FIGURE 4:**
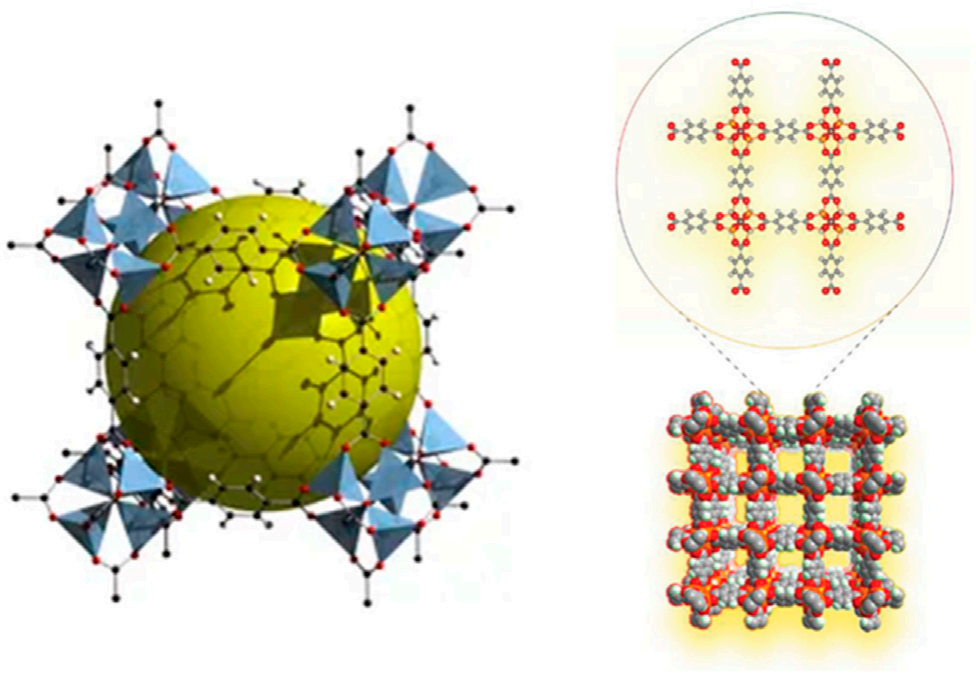
MOF-5 three-dimensional structure diagram (Li et al., 1999; Xie et al., 2022).

Although zeolite molecular sieves have a high specific surface area and uniform pore structure, their poor framework controllability and difficult control during the synthesis process restrict their development. In order to make up for its shortcomings and play its strengths, after continuous exploration and research, a major challenge has been completed in the synthesis of solid crystalline materials. Without changing the topological structure of the material, the chemical composition, functionality, and pore size of the material are systematically controlled. Yaghi’s research group first proposed the concept of MOFs and synthesized the landmark new porous material MOF-5 (Figure 4) (Li et al., 1999). This type of porous material connects metal ions and organic carboxylate ligands through coordination bonds to form a network structure. They have a high specific surface area and porosity, excellent controllability, and easy functionalization (Shen et al., 2021a). By selecting different metal ions and a variety of organic ligands, adjusting the ratio between the metal and the ligand, and using appropriate synthesis methods, MOFs with varying pore characteristics can be obtained. The research group represented by Yaghi (Li et al., 1999; Furukawa et al., 2010) systematically studied the structure of MOFs and their CO_2_ adsorption behavior. Among them, the M-MOF-74 series (Caskey et al., 2008) has superior selective adsorption capacity for CO_2_; Williams et al. The adsorption capacity of HKUST-l [38] for CO_2_ under normal temperature and pressure can reach 4.1 mmol/g. Matzger et al. prepared Mg-MOF-77 with a two-dimensional hexagonal pore structure and exposed metal nodes. The gas adsorption test showed that under 296 K and 1bar conditions, the saturated adsorption capacity of CO_2_ reached 8.0 mmol/g (Chui et al., 1999). Britt et al. tested the CO_2_ adsorption and separation performance of Mg-MOF-77 under dynamic breakthrough conditions, and found that the saturated adsorption capacity still reached 2.0 mmol/g, and showed excellent CO_2_/CH_4_ separation performance (Britt et al., 2009). In recent years, people have conducted continuous research and improvement on the synthesis method of MOFs. Currently, the procedures for synthesizing MOFs include the solvent method (Zulys et al., 2020), microwave synthesis method (Jhung et al., 2005), and reagent-free-mechanochemical synthesis method (Pichon et al., 2006). MOFs’ rich and diverse structure makes these materials widely used in gas adsorption and storage, separation, catalysis, and sensing (Rosi et al., 2003; Horcajada et al., 2010; Liu et al., 2012; Liu et al., 2014; Cadiau et al., 2016). Among the many reported MOFs, the metal-organic framework material MOF-74, due to its strong adsorption force between the unsaturated metal sites and CO_2_, is currently a hot spot in the field of CO_2_ adsorption and separation (among which Mg-MOF-74, the CO_2_ adsorption capacity at 298 K and 1bar is as high as 8.3 mmol/g (Caskey et al., 2008). Table 5 compares the CO_2_ adsorption performance of the more common metal-organic framework materials under low pressure. It can be seen that the metal organic framework material Mg-MOF-74 has better CO_2_ adsorption performance under low pressure.

**TABLE 5:** Comparison of CO_2_ adsorption performance of common adsorbent materials under low pressure (Li et al., 2020b).

Material	BET (m^2^/g)	Langmuir (m^2^/g)	Capacity (wt%)	Pressure (bar)	Temp (K)	References
Mg-MOF-74	1174	1733	27.5	1	298	Bao et al., (2011)
			27.2	1	298	Yazaydin et al., (2009)
	1800	2060	26.7	1	298	Mason et al., (2011)
	1495	1905	26	1	296	Caskey et al., (2008)
Co-MOF-74	957	1388	24.9	1	298	Yazaydin et al., (2009)
Ni-MOF-74	936	1356	23.9	1	298	Yazaydin et al., (2009)
Zn-MOF-74			19.8	1	296	Yazaydin et al., (2009)
HKUST-1		1492	18.4	1	298	Yazaydin et al., (2009)

The pore size of the mesoporous silica material is between 2 nm and 50 nm. Mesoporous silica materials mainly include silica nanoparticles, silica hollow spheres, silica nanotubes, mesoporous silica foams and aerogels. Mesoporous silica has the advantages of high specific surface area, large pore volume, narrow pore size distribution, and good regeneration stability. It has also made significant progress in CO_2_ capture The widely used mesoporous silica (Sharp et al., 2021), including MCM-41, SBA-15 and KIT-6 series, can successfully separate CO_2_ from the mixture of CH_4_ and N_2_. However, the hydrothermal stability of mesoporous silica is low, which is due to the easy hydrolysis of Si-O-Si in the presence of water vapor at high temperatures (Rafigh and Heydarinasab, 2017). Zhang et al. (Zhang et al., 2019) used the silicate supernatant extracted from the alkali melt as the raw material, and used the addition polymer of polypropylene glycol and ethylene oxide (polyether) P123 and trimethylbenzene (TMB) was used as the structure orientation. Agent and swelling agent, mesoporous silica foam materials were prepared under acidic conditions. The study found that the CO_2_ adsorption capacity can reach 4.7 mmol/g at 75°C and 1bar.

### 3.2 Adsorption Selectivity

The CO_2_ molecule has a large quadrupole moment and a high polarization rate (29.11 × 10^−25^ cm^3^), so the polar surface of the porous adsorbent has a strong inducing effect on CO_2_ and has a large affinity. With the deepening of the understanding of the structure and chemical properties of ZIFs materials, researchers began to explore the relationship between CO_2_ adsorption and separation performance and structure. Banerjee et al. (Banerjee et al., 2009) used a series of materials with different pore structure parameters and surface polarities (ZIF^−^68, ^−^69, ^−^70, ^−^78, ^−^79, ^−^80, ^−^81, ^−^82) as the research object. The effects of pore size, specific surface area and surface groups on the CO_2_ adsorption capacity and selectivity were analyzed. They found that the amount of CO_2_ adsorbed at low pressure is directly related to the polarity of the surface groups. ZIF-78 (^−^NO_2_) and ZIF-82 (^−^CN) with strong polar groups show better selectivity than other ZIFs. This is because the strongly polar group exhibits a strong dipole motion, which has a stronger dipole quadrupole interaction with the CO_2_ molecule, thereby enhancing the affinity for the pair and exhibiting better selectivity. Morris et al. (Morris et al., 2010) conducted experiments and grand canonical Monte Carlo (GCMC) simulations on a set of ZIFs with the same topological structure. The two methods verified the surface polarity and the configuration of nitrogen-containing groups and the electrons between CO_2_ molecules. The attraction has a decisive effect on the adsorption capacity of low-pressure CO_2_.

**FIGURE 5:**
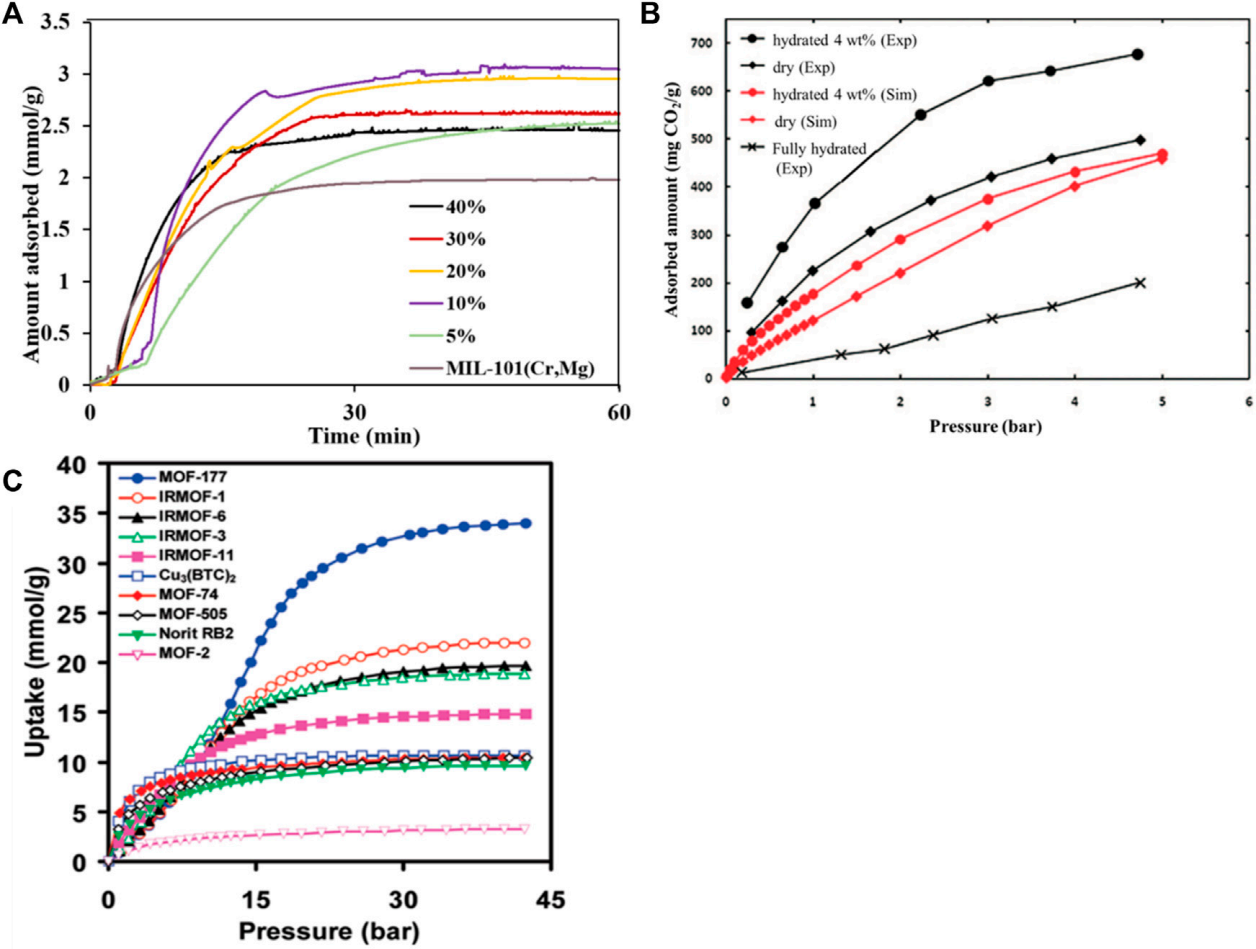
The selective absorption of CO_2_ by MOFs: **(A)** the effect of PEI loading on the adsorption selectivity of MIL-101 (Cr, Mg) adsorbent (Gaikwad et al., 2019); **(B)**the adsorption selectivity of HKUST-1 for CO_2_/H_2_O (Yazaydın et al., 2009) (**(C)** the adsorption properties of MOF-177 and Cu-BTC under different pressures (Millward and Yaghi, 2005).

Traditional physical adsorbents such as activated carbon and molecular sieves have small adsorption capacity, low selectivity, and sensitivity to adsorption temperature. MOFs have the characteristics of good selectivity, easy regeneration, high thermal and chemical stability, large specific surface (up to 7000 m^2^/g), large pore volume (55–90%), and low density (0.21–1 g/cm^3^)). Their pore structure is regular and adjustable, and the organic ligands can be modified to design and synthesize crystalline materials with specific physical properties and chemical functions (Li et al., 2009). Gaikwad et al. (Gaikwad et al., 2019) synthesized a series of MIL-101 (Cr, Mg) adsorbents with different polyethyleneimine (PEI) loadings, as shown in Figure 5A. The study found that although the specific surface area and total pore volume of MIL-101 (Cr, Mg) decreased significantly with the increase of PEI loading, the MIL-101 (Cr, Mg) adsorbent showed significant performance under low pressure (Giménez-Marqués et al., 2019; Chanut et al., 2020). At 25°C and 1bar, when the PEI loading is 20 wt%, the CO_2_ adsorption capacity of the adsorbent can reach 3 mmol/g. It is generally believed that in water vapor, the CO_2_ adsorption capacity and selectivity of the metal-organic framework will decrease because water molecules will compete with CO_2_ for adsorption sites (Liu et al., 2010). However, Snurr et al. (Yazaydın et al., 2009) synthesized a new type of metal-organic framework HKUST-1, and performed CO_2_ adsorption experiments on it when the mixed flue gas contained 4 vol% water vapor. As shown in Figure 5B, it is found that unlike other metal-organic frameworks, water vapor increases the CO_2_ adsorption capacity. Millward et al. (Millward and Yaghi, 2005) reported that under a pressure of 3.5 MPa, with MOF-177 as the adsorbent, the highest carbon dioxide adsorption capacity was as high as 33.5 mmol/g, as shown in Figure 5C. Subsequently, the water molecules and the open metal sites of the framework material are coordinated and complex. Therefore, the selectivity of the Cu-BTC organic framework material to CO_2_ is significantly improved. The current research results show that metal organic frameworks can adsorb carbon dioxide with a strong quadrupole moment by selecting metal clusters with strong interactions with carbon dioxide or selecting different nodes to construct different pore sizes. In addition, the CO_2_ adsorption capacity of MOFs can be increased by modification. However, the adsorption capacity is very low under high temperature and humidity, which limits its industrial application. To obtain a metal framework with a high adsorption capacity in a humid environment, further research and exploration are needed.

**FIGURE 6:**
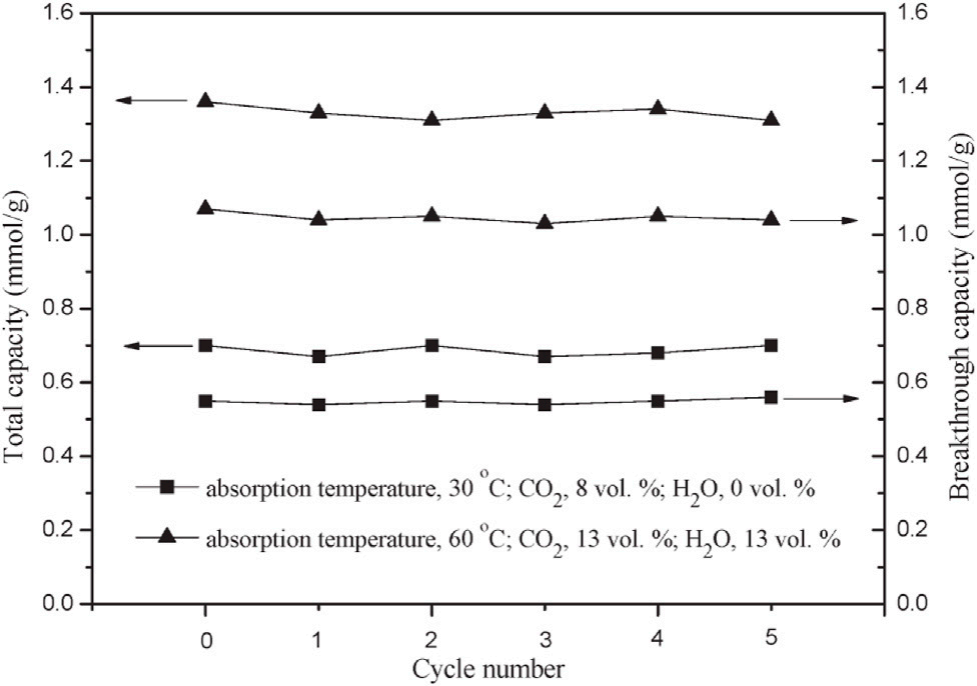
Carbon dioxide’s total capacity and breakthrough capacity on the MgO/Al_2_O_3_ adsorbent in the presence and absence of water vapor in multiple absorption/desorption cycles (Li et al., 2010). The pressure is 1 bar, the flow rate is 80 ml/min, the balance gas is N_2_, the regeneration temperature is 350°C, and the amount of MgO/Al_2_O_3_ adsorbent is 7.2 g.

Metal oxides separate carbon dioxide from mixed gases by reacting with CO_2_ molecules. Common metal oxide materials mainly include calcium oxide, magnesium oxide, lithium zirconate/lithium silicate, and other materials. Carbon dioxide is an acid gas, which is easier to adsorb on the basic sites of metal oxides. Therefore, metal oxide adsorbents have high adsorption capacity, good selectivity, vast sources, and low cost. MgO and CaO are alkaline adsorbents, and their adsorption mechanism is usually the acid-base neutralization reaction with the acid gas CO_2_ to form carbonate (Li et al., 2005; Shen and Zhang, 2020). Among them, the MgO-based adsorbent has a higher theoretical adsorption capacity, about 1.1 g⋅g^−1^, and is considered an ideal medium temperature CO_2_ adsorbent. However, in practical applications, the adsorption rate of MgO-based adsorbents is slow, the actual adsorption capacity is low, generally lower than 0.01 g⋅g^−1^, and the cycle stability is poor. These problems limit the industrial use of MgO-based adsorbents. The extensive application of the above (Wang et al., 2011). In order to increase the adsorption capacity of MgO-based adsorbents, researchers used a loading method with porous alumina, activated carbon, and mesoporous silica as carriers (Gu et al., 2010; Han et al., 2014; Hanif et al., 2016) to increase the adsorption capacity to 0.085 g⋅g^−1^. For example, CaO reacts with CO_2_ to form CaCO_3_ under certain conditions. This method fixes CO_2_ through chemical reaction has the advantages of high selectivity and large adsorption capacity, so it has quickly become a research hotspot. Li et al. (Li et al., 2010) prepared MgO/Al_2_O_3_ adsorbent and studied its performance in low-temperature capture of CO_2_ on a fixed bed. The results show that when the MgO loading is 10 wt%, the MgO/Al_2_O_3_ adsorbent has the largest CO_2_ adsorption capacity. And as the water vapor concentration increases, the CO_2_ capture capacity of the adsorbent first increases and then decreases. As shown in Figure 6, when the adsorption temperature is 60°C and the concentration of CO_2_ and water vapor are 13 vol%, the CO_2_ adsorption capacity of the adsorbent reaches its peak value, which is 1.36 mmol/g. In addition, after five regeneration experiments, it was found that the adsorption performance of the adsorbent remained stable. However, metal oxides as CO_2_ capture materials also have obvious defects, such as high adsorption temperature (the adsorption temperature of CaO is above 450°C (Nie et al., 2017), low adsorption capacity (the adsorption capacity of CO_2_ is 0.5 mmol/g at 450°C and 20 bar (Hassanzadeh and Abbasian, 2010)), and slow adsorption rate [the lithium-based oxide needs 2 days to reach adsorption saturation at 600 °C (Ida et al., 2004)]. The high regeneration energy consumption is high [the degassing temperature is above 250°C (Harada et al., 2015)]. In short, a chemical adsorbent that uses a chemical reaction between adsorbent and carbon dioxide has the advantages of large CO_2_ adsorption capacity and good selectivity to carbon dioxide, but it also has the disadvantages of difficult desorption and high energy consumption for regeneration.

**FIGURE 7:**
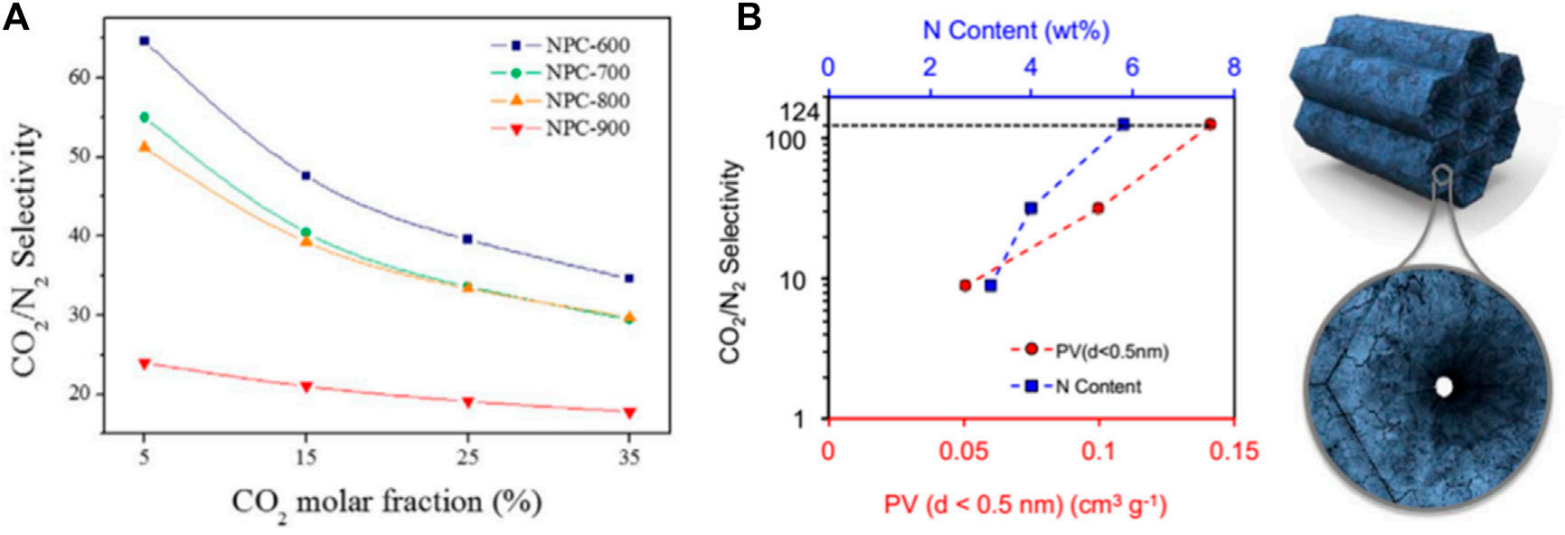
Effect of nitrogen modified carbon-based adsorbent on CO_2_ selectivity: **(A)** Nitrogen-containing pitch-based activated carbon (NPC) selectivity as a function of CO_2_ mole fraction at 298 K and 1 bar total pressure (Lee et al., 2016); **(B)** The correlation between CO_2_/N_2_ selectivity and ultra-small pore volume and the correlation between CO_2_/N_2_ selectivity and N content (To et al., 2016).

The polar surface of the CO_2_ adsorption material is another important factor that affects the CO_2_ adsorption performance, especially the type and quantity of nitrogen-containing groups in the carbonaceous adsorption material. Surface functionalization methods of porous carbon materials include two types (Li et al., 2019; Li et al., 2020c): 1) utilization of functionalized precursors (such as nitrogen-rich compounds) to obtain heteroatom-doped porous carbon in one step and 2) post-treatment to introduce specific functional groups. In terms of stability, the former has outstanding advantages. The surface modified by nitrogen functional groups have a solid inducing effect on CO_2_ molecules, and strong interaction with them, which enhances the selective recognition of CO_2_ molecules and improves the CO_2_ adsorption capacity of the material. Lee et al. (Lee et al., 2016) discussed the effect of nitrogen content on the CO_2_/N_2_ separation performance of pitch-based porous carbon, as shown in Figure 7A. Studies have shown that the interaction between strong acids leads to the presence of N elements that is beneficial to CO_2_ adsorption, and the decrease in nitrogen content will increase the hydrophobicity of N_2_. Improve the selectivity of CO_2_/N_2_. Jennifer and Bao et al. (To et al., 2016) studied the effects of different nitrogen chemical states on the CO_2_ adsorption capacity and CO_2_/N_2_ selectivity of polypyrrole-based carbons, and confirmed that pyrrolic N-5 and pyridonic N-5′ are most conducive to the improvement of CO_2_ adsorption performance, as shown in Figure 7B. Govind Sethia also got a similar conclusion (Sethia and Sayari, 2015), the ultra-microporous doped nitrogen plays an important role in CO_2_ adsorption. Doping basic or electron-rich heteroatoms, such as nitrogen, into activated porous carbon frameworks effectively improves the CO_2_ uptake capacity of adsorbents. Because there are abundant basic sites on the surface that can act as anchors to capture weakly acidic CO_2_ molecules. In addition, selecting different precursors (such as fruit shell, organic matter, coal-based, etc. ) to prepare porous carbon materials with high specific surface area and rich pore structure can effectively adsorb carbon dioxide molecules in the adsorbent. Using natural plants as raw materials, it has low cost, high electrochemical performance and certain adsorption, and has broad application prospects in the fields of energy and environmental protection. However, the adsorption of CO_2_ by porous carbon materials is physically-interacted primarily, and the adsorption strength is weak. Therefore, the adsorption performance is sensitive to temperature, and the selectivity is poor. The development of porous carbons with high selectivity and high adsorption capacity or the selection of highly active materials for composites is still the focus of future research.

### 3.3 Adsorption Stability

**FIGURE 8:**
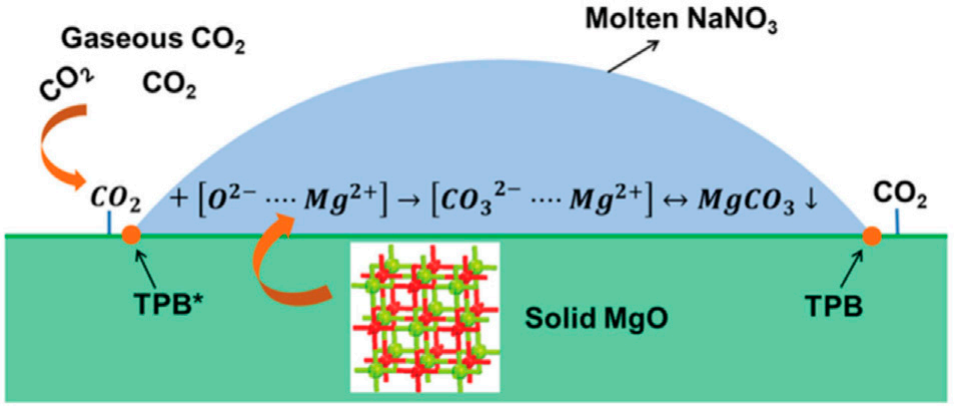
Molten NaNO_3_ promotes the CO_2_ adsorption mechanism of MgO-based adsorbents (Zhang et al., 2014).

In practical applications, an essential condition for measuring the performance of an adsorption material is its cyclic adsorption performance. After many times of adsorption and desorption, its adsorption performance should not be significantly degraded (Liu et al., 2021). According to analysis, the alkali metal salt is in a molten state at high temperature (350–400°C). It is easy to flow in a wide range, resulting in poor cycle stability of the adsorbent. However, the main challenge of MgO-based adsorbents is that due to their high (Mg^2+^ - O^2-^) lattice energy, the actual CO_2_ adsorption capacity of MgO is very low, and the CO_2_ adsorption rate is poor. In recent years, studies have shown that modifying MgO with alkali metal carbonate or nitrate can effectively improve its CO_2_ capture performance. Since the alkali metal nitrate is in a molten state in the medium temperature range (250–500°C), the reason why it promotes the adsorption performance of the MgO-based adsorbent mainly lies in the following two aspects: First, the CO_2_ in the gas phase is in the molten alkali metal nitrate. There is a certain degree of solubility in nitrate, which can be converted into $CO_{3}^{2-}$ by O^2-^ in nitrate; second, molten alkali metal nitrate can dissolve a certain degree of MgO, which will have high bond energy (Mg^2+^ - O^2-^) dissociates into (Mg^2+^ ... O^2-^) ion pair. Therefore, the molten alkali metal salt can transfer the gas-solid reaction interface of MgO and CO_2_ to the molten phase for dissolution and nucleation growth processes, thereby increasing the adsorption rate and adsorption capacity of the MgO-based adsorbent material. Zhang et al. (Zhang et al., 2014) studied the promoting effect and mechanism of molten alkali metal nitrate on the adsorption of CO_2_ by MgO. The results show that the conversion rate of MgO is significantly increased under the promotion of molten NaNO_3_. In order to verify the promoting effect of molten nitrate on the adsorption of CO_2_ by MgO, they investigated the effect of mixed nitrates with different melting points on the adsorption performance of MgO. The results showed that when the adsorption temperature is higher than the melting point of the mixed nitrate, molten nitrate can promote the adsorption of CO_2_ by MgO. They believe that molten nitrate can dissolve part of MgO, thereby reducing the lattice energy of MgO. MgO exists in the form of (Mg^2+^ ... O^2-^) ion pairs in the molten salt, and then reacts with CO_2_ to generate MgCO_3_. They pointed out that the main place of carbonation reaction in MgO is at the triple point of molten alkali metal nitrate, gas-phase CO_2_ and solid-phase MgO, as shown in Figure 8.

**FIGURE 9:**
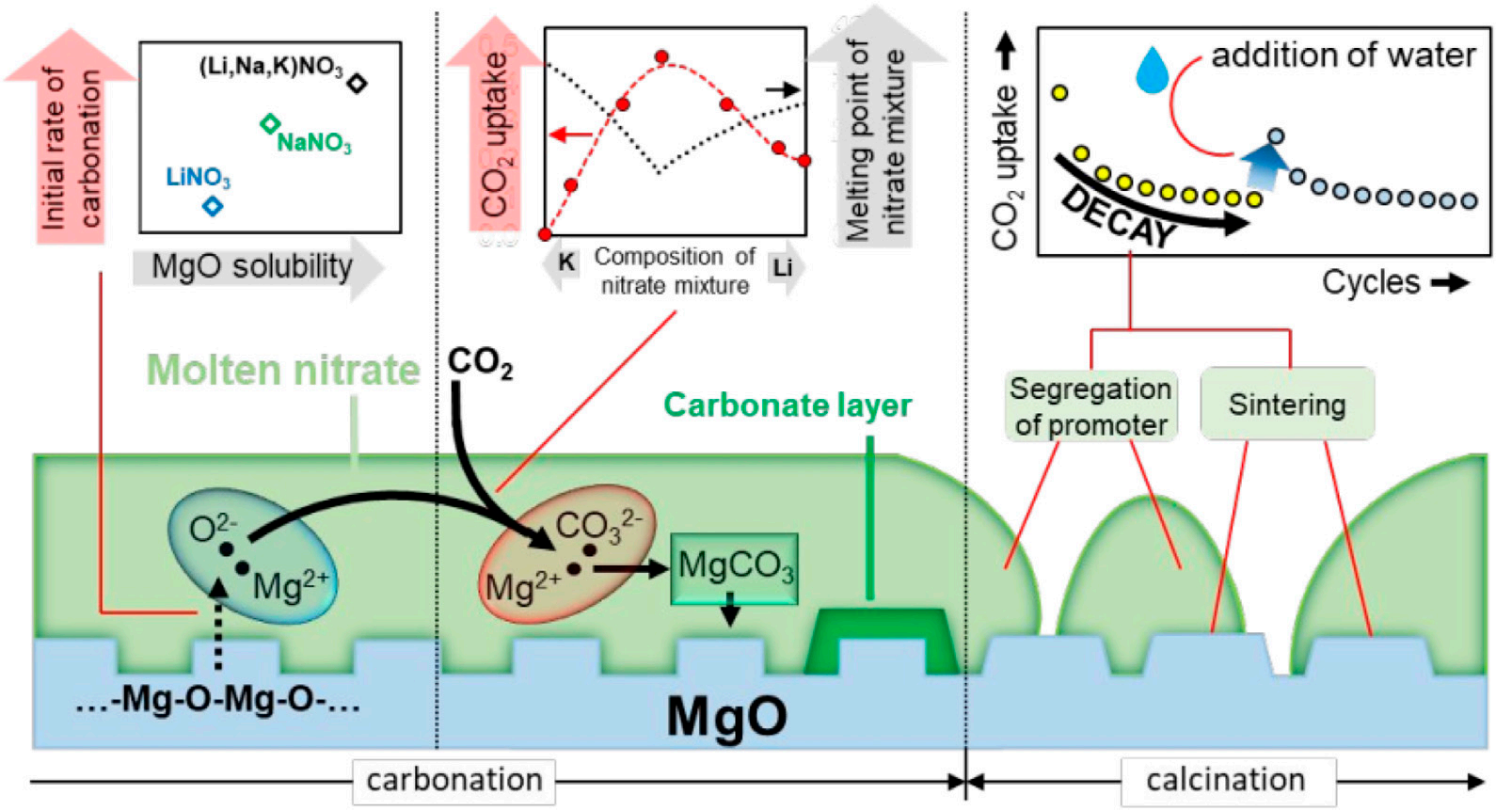
Molten alkali metal nitrate promotes CO_2_ adsorption by MgO-based adsorbents (Dal Pozzo et al., 2019).

Pozzo et al. (Dal Pozzo et al., 2019) studied the mechanism of molten nitrate promoting the adsorption performance of MgO and clarified the reason for the deactivation of the adsorbent material for a long time. Studies have pointed out that the promotion of MgO by molten NaNO_3_ is mainly the dissolution of MgO in molten alkali metal nitrate, promoting the faster formation of Mg^2+^. Therefore, the dissolution of MgO in molten alkali metal nitrate is the decisive step in adsorption, as shown in Figure 9. In addition, studies have shown that the adsorption capacity of molten NaNO_3_ modified MgO adsorbents gradually decreases during the cycle, which is mainly due to the aggregation of molten alkali metal nitrates, resulting in a decrease in the active surface. Based on this, they proposed a method of activating the adsorbent material, the deactivated material is re-dissolved in water and then dried. The alkali metal nitrate on the surface of the adsorbent material is redistributed, and its adsorption performance is also improved.

Regarding the stability of porous carbon adsorbents, studies have found that the type and number of nitrogen-containing groups in porous carbon will be affected by the pyrolysis temperature. With the increase in pyrolysis temperature, the number of nitrogen-containing active sites gradually decreased. The nitrogen-containing groups gradually changed from pyrrolic N-5 and pyridonic N-5′ to Quatemary-N (To et al., 2016; Cai et al., 2021). Commonly used nitrogen introducing agents include ammonia, urea, organic amine solutions, etc [65], covering three modification methods of gas, solid and liquid. Przepiórski et al. (Przepiórski et al., 2004) used commercial activated carbon as a raw material, fed ammonia gas at 200–1000 °C and kept it for 2 h to prepare nitrogen-doped activated carbon. The study found that the activated carbon after ammonia heat treatment has stronger CO_2_ adsorption performance than commercial activated carbon, confirming the effectiveness of ammonia nitrogen incorporation; the adsorbent prepared at 400°C has the largest CO_2_ adsorption capacity; The poor adsorption performance of samples activated at temperatures above 400°C may be due to the nitrogen-containing functional groups blocking the micropores or changing the pore structure (Shen et al., 2021b). Many scholars have studied the formation mechanism of nitrogen-containing functional groups during high-temperature ammonia treatment. Studies have shown that ammonia gas will decompose into free radicals at high temperatures, such as NH_2_, NH, hydrogen atoms, and nitrogen atoms. These free radicals are accelerated by Brownian motion at high temperatures and continually collide with carbon atoms to form nitrogen-containing functional groups (Bota and Abotsi, 1994). Karimi et al. (Karimi et al., 2018) mixed commercial activated carbon with urea and activated it at a high temperature of 800°C. The study found that the activated carbon doped with nitrogen via urea has a larger CO_2_ adsorption capacity than other samples. Keramati et al. (Keramati and Ghoreyshi, 2014) modified commercial activated carbon by immersion in a triethylenetetramine (TETA) solution. The results showed that the amine functionalization of activated carbon significantly improved its CO_2_ adsorption performance. Under 25°C and 40bar conditions, the maximum CO_2_ adsorption capacity of the activated carbon adsorbent impregnated with organic amine solution is as high as 16.16 mmol/g, which is 90% higher than the adsorption capacity of the original commercial activated carbon. Various studies have confirmed that urea and organic amine solution doped with nitrogen can significantly improve the common activated carbon adsorbent.

As for MOF materials, the framework of MOFs is formed by connecting metal ions and ligands through coordination bonds. This coordination bond is easily damaged under harsh conditions such as high temperature, humidity, and acid-base environment, resulting in the collapse of the entire skeleton. Metal-organic framework materials Ni-MOF-74 and Co-MOF-74 have higher water stability and hydrophobicity than Mg-MOF-74. Yang et al. (Jiao et al., 2015) compared Mg-MOF-74 doped with Co., or Ni metal through metal doping, compared the effects of doping metal type and doping ratio on the performance of MOF-74, and finally found that when doped with Ni content of 16%, the water stability of the Ni&Mg-MOF-74 framework containing heterogeneous metals is maximized. However, there are few works about the CO_2_ adsorption performance of metal-doped MOF-74, especially the CO_2_ adsorption performance under humid conditions. In 2016, Zhai et al. (Zhai et al., 2016) studied the regulation effects of heterogeneous metals Sc/Mg and V/Mg on the adsorption performance and heat of adsorption of MOF-74. The study found that MOF-74 with V and Mg as the metal center is at 273 K. Its CO_2_ adsorption performance can reach 207.6 cm^3^/g under the condition of 1bar. In 2017, Joshua et al. (Howe et al., 2017) further studied the effect of Ni or Cd doping on the stability of Mg-MOF-74, and pointed out that the MO bond on the top of M-MOF-74 is the key to the water stability of the material. Among all MO bonds, the top MO bond is the most unstable and most susceptible to the influence of H_2_O molecules. The doping of metals brings about asymmetric defects in the lattice structure of the skeleton, thereby bringing about contraction of the top M-O bond, enhancing the stability of the M-O bond, and further enhancing the water stability of the skeleton.

Modification of MOF-74 after synthesis is currently the most widely reported modification method in the literature. Chemical solution impregnation is the most common and effective way, such as ammonia impregnation and ethylenediamine solution impregnation. Cao et al. (Cao et al., 2013) used different mass fractions of tetraethylpentamine (TEPA) to modify the metal-organic framework Mg-MOF-74, and explored its effect on CO_2_ adsorption performance. The breakthrough curve obtained by the test shows that the CO_2_ adsorption capacity of TEPA-Mg-MOF-74 with appropriate modification amount is increased from 2.67 mmol/g to 6.06 mmol/g compared with Mg-MOF-74, and the CO_2_ adsorption capacity under humid conditions further increase to 8.31 mmol/g. The cycle stability test showed that the CO_2_ adsorption capacity of TEPA-Mg-MOF-74 decreased by only 3% after five cycles of absorption and desorption, which indicated that the amino-modified Mg-MOF-74 is a better method after synthesis. Choi et al. (Choi et al., 2012) used ethanediamine (ED) solution to modify Mg-MOF-74 after synthesis. After modification, each unit cell of ED-Mg-MOF-74 contained an ethylenediamine molecule. After four cycles of absorption/desorption, the stability and regeneration capacity of the material are significantly improved, and the CO_2_ adsorption capacity of the framework material when the CO_2_ partial pressure is less than 400 ppm (298 K) is also reduced from 1.35 mmol/g before modification, slightly increased to 1.51 mmol/g. In addition, Mcdonald et al. (McDonald et al., 2012) successfully applied the post-synthesis modification method to introduce the dimethylethylenediamine (mmen) group into Mg-MOF-74. They found that the modified mmen-Mg-MOF-74 It has a very high CO_2_ adsorption capacity under low pressure. At 0.39 mba and 298 K, the CO_2_ adsorption capacity is 2.38 mmol/g (9.5 wt%), and its isometric heat of adsorption reaches -74 kJ/mol, showing strong adsorption force. In 2017, Su et al. (Su et al., 2017) were based on solution immersion modification and used macromolecular tetraethylpentamine (TEPA) to modify the MOF material Mg-MOF-74 after synthesis. The study found that the TEPA modification combined the framework to the single the adsorption capacity of component CO_2_ increased from 23.4 wt% to 26.9 wt%. In addition, the CO_2_ adsorption capacity and adsorption stability of the framework under humid conditions (CO_2_/H_2_O mixed gas) have also been significantly improved. It shows that the synergistic effect of the amine functional group and the metal center has a positive effect on the CO_2_ adsorption capacity of the framework. Although most of the post-synthesis modification studies focus on different kinds of ammonia solution immersion modification, there are other methods. For example, in 2015, Fernandez et al. (Fernandez et al., 2015) impregnated the metal organic framework material Ni-MOF-74 with a chloroform solution (P123), and introduced the hydrophobic group P123 to the outer surface of the Ni-MOF-74 pores by adsorption. The original CO_2_ adsorption capacity of the framework is retained. In addition, the water stability of the material is significantly improved, and the H_2_O adsorption capacity is reduced by three times.

In addition to NO_X_, the flue gas also contains impurities such as water vapor, SO_X_, O_X,_ and heavy metals. If the adsorbent is not tolerant to these impurities, the overall economics of the CO_2_ separation process will increase. In general, moisture will adversely affect the CO_2_ adsorption process of various physical sorbents. The vast majority of reports on physical sorbents have not studied the effect of humidity, so the technology to absorb CO_2_ from flue gas may include an upstream drying step. It is generally believed that CO_2_ adsorbents have a high affinity for NO_X_ and SO_X_, which may adversely affect the CO_2_ adsorption capacity of the material. Therefore, in most cases, NO_X_ and SO_2_ need to be removed from the flue gas before CO_2_ capture.

## 4 Summary and Outlook

**FIGURE 10:**
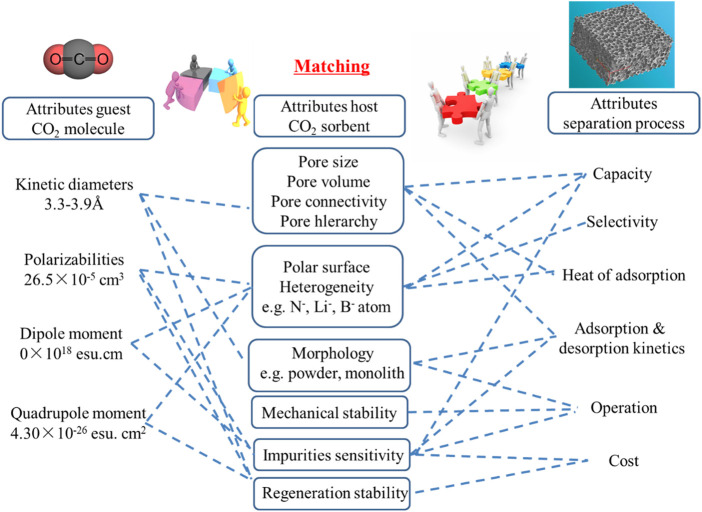
Design criteria for high-performance absorbent materials.

Comprehensive literature reports show that the design of high-efficiency CO_2_ adsorption materials must match the characteristics of the application object and meet the requirements of the application process. The essential features of CO_2_ molecules are small dynamic size (approximately 3.3 Å) and electric quadrupole properties ${(}^{\delta -}C{=}^{\delta +}C^{\delta +}=O^{\delta -})$ compared with other gases, CO_2_ molecular polarizability is higher. The CO_2_ volume concentration of the gas source after combustion is usually less than 15%, the total pressure is about 1 atm, and other components include N_2_, H_2_O, etc., The adsorption and separation of this gas source are the most difficult because the total pressure is not high, the volume flow rate is large, and the CO_2_ concentration (partial pressure) is low. Abundant micropores, high specific surface area, and adjustable surface chemistry are ideal adsorbent characteristics in terms of matching dynamic size and polarizability of CO_2_ molecules. At the same time, the porous adsorption material has excellent chemical stability and thermal stability (inert atmosphere). It has obvious advantages when applied to the separation of complex gas sources, which is also a key consideration. However, the current porous adsorbent materials are obviously insufficient in macro and micro structure control, especially in pore structure control and surface chemical modulation. The macroscopic and microscopic structural parameters of porous adsorbent materials were analyzed, and the corresponding design and preparation strategies were summarized. It is urgent to make a breakthrough in pore structure regulation while maintaining the advantages of porous adsorbent materials with developed pores and excellent stability. From the perspective of adsorption kinetics, eliminating internal and external diffusion is the key. As shown in Figure 10, effective measures include constructing multi-level pores, introducing mesopores, and reducing the size of material structural units in any dimension, representing current hot issues in the structural design of CO_2_ adsorption porous materials.

In short, in principle, the physical adsorption process is mainly based on the intermolecular attraction between the guest molecules and the active points on the surface of the porous solid adsorbent, which is a surface process. From the perspective of adsorption effect, large adsorption capacity, high selectivity, fast adsorption kinetics and excellent cycle stability are the key parameters for maintaining high performance in adsorption separation. Efficient adsorption materials should have abundant micropores (storage space), specific surface chemistry (with strong interaction), short diffusion paths (multi-level pores, small-scale structural units) and excellent structural stability (mechanical properties) good). As mentioned earlier, post-combustion carbon capture technology has the following characteristics: relatively low CO_2_ partial pressure (3–20 vol%), contains water vapor (1–10 vol%) (White et al., 2003; Demessence et al., 2009), large volume flow, etc., (Yazaydin et al., 2009; Plaza et al., 2010) These conditions require the adsorption material to have strong adsorption capacity, resistance to water vapor and good mechanical properties (wear resistance and powdering resistance) under dynamic low partial pressure conditions.

The monolithic material is a structural material in which the internal framework and the pores are continuous. Common monolithic materials include cordierite, cinnamon dioxide, polymers, and porous carbon materials. They have the following characteristics:

1) The design is flexible, easy to operate, and can meet the needs of the macroscopic appearance of the application.
2) The staggered skeleton and pores form an isotropic microstructure, which can ensure the uniform diffusion of the fluid in all directions;
3) The mass transfer resistance is small, the contact efficiency is high, and the penetration is quick. One of the current research hotspots is designing and preparing functional monolithic porous carbon, which includes explicitly developing a new type of polymerization system or a new type of carbon source, precise control of the pore structure, and surface-oriented functionalization. The design and realization of a series of multi-stage pores is the primary goal of precise pore structure control. CO_2_ adsorption separation is a process of fluid transmission, diffusion, and storage. The macropores and mesopores in adsorption materials function as the channels for rapid CO_2_ transmission in this process, ensuring rapid adsorption kinetics. The abundant micropores of the adsorption material can be used as the CO_2_ storage place ensuring high adsorption capacity. In nature, organisms, ranging from towering trees to small system organizations, generally use multi-level structures as organizational frameworks to complete their life processes, which involve fluid diffusion, transmission, and storage, such as the transmission of water and nutrients in plants. After thousands of years of evolution of the survival of the fittest, the multi-level structure is still the main structural organization form, indicating that the structure has certain advantages, which is also the inspiration for researchers from nature. Given the advantages and disadvantages of various adsorption materials’ structure and performance, as well as inspired by the structure and organization of natural organisms and their fluid transportation and diffusion behavior, it can be concluded that the multi-level pore monolithic structure is an ideal organization structure.

Although porous carbon materials have achieved good adsorption and separation effects, most of them are powder samples, and there are usually problems such as pore blockage during the molding process (Williams, 2001; El Kadib et al., 2009; Lohe et al., 2009). Under the dynamic impact of airflow, the pulverization is severe, which leads to the reduction of adsorption and separation efficiency [99–101]. In addition, most of the currently reported adsorption and separation studies are limited to equilibrium adsorption, which is far from the dynamic penetration conditions in practical applications. In summary, we believe that developing a monolithic material preparation method with a controllable structure and good mechanical properties and the static equilibrium adsorption and dynamic penetration and separation of the obtained adsorbent materials is the areas where current research needs to be strengthened. Based on maintaining the adsorption capacity of the material, improving its mechanical properties is conducive to improving the structural stability of the material during the adsorption process; the comprehensive study of equilibrium adsorption and dynamic penetration can deepen the understanding of the dynamic adsorption separation process. Considering the characteristics of high flow rate and high impact of the air source, the material also needs to have a certain degree of mechanical strength. Most of the current research is focused on improving the adsorption capacity of porous materials, and significant progress has been made. However, the stability of the material structure, especially the stability of the adsorption and separation cycle under dynamic conditions, has not attracted enough attention. Therefore, research on the mechanical strength and the cycle performance in adsorption and separation also needs to be strengthened.

This review discusses the current research progress of porous adsorption materials from the perspective of industrial flue gas carbon capture. After comparing a variety of carbon adsorption materials, including carbon-based materials, zeolites, metal organic framework materials, and metal oxides, it can be found that different adsorbents immobilize CO_2_ under very different temperatures, pressures, carbon dioxide concentrations, and relative humidity of the gas. Therefore, the choice of adsorbent needs to be determined according to the actual application. The ideal adsorbent needs to meet the following requirements: the higher the carbon dioxide adsorption capacity, the higher the adsorption and desorption rate, the better the cycle stability, the better the mechanical strength, and the lower the preparation cost. There are currently no suitable adsorbent materials to meet the above requirements, and each material has its inherent advantages and disadvantages. When selecting adsorbents, the advantages and disadvantages of various materials should be comprehensively analyzed, and the lowest-cost adsorbent material that meets the CO_2_ capture requirements should be chosen according to the actual working conditions.
