# Erratum: Porous adsorption materials for carbon dioxide capture in industrial flue gas

**DOI:** 10.3389/fchem.2023.1182159

**Published:** 2023-03-17

**Authors:** Frontiers Production Office

**Affiliations:** Frontiers Media SA, Lausanne, Switzerland

**Keywords:** carbon capture and storage, porous adsorption materials, industrial waste gas, adsorption mechanism, carbon dioxide

An omission to the **Funding** section of the original article was made in error. The following sentence has been added: “Open access funding was provided by Empa - Swiss Federal Laboratories For Materials Science And Technology.”

The original version of this article has been updated.

